# Human Co-Infection with Avian and Seasonal Influenza Viruses, China

**DOI:** 10.3201/eid2011.140897

**Published:** 2014-11

**Authors:** Jun Li, Yu Kou, Xinfen Yu, Yongxiang Sun, Yinyan Zhou, Xiaoying Pu, Tao Jin, Jingcao Pan, George F. Gao

**Affiliations:** Hangzhou Center for Disease Control and Prevention, Hangzhou, China (J. Li, Y. Kou, X. Yu, Y. Zhou, X. Pu, J. Pan);; Xiaoshan District Center for Disease Control and Prevention, Hangzhou (Y. Sun);; BGI-Shenzhen, Shenzhen, China (T. Jin);; Chinese Academy of Sciences Key Laboratory of Pathogenic Microbiology and Immunology, Beijing, China (G.F. Gao)

**Keywords:** influenza A(H7N9) virus, influenza A(H1N1)pdm09 virus, influenza B virus, co-infection, influenza, viruses, China

**To the Editor:** In April 2013, a case of co-infection with avian-origin influenza A(H7N9) virus and seasonal influenza A(H3N2) virus was reported in Jiangsu Province, China (*1*). This case raised concern over the possible occurrence of new reassortants with enhanced transmissibility among humans. Because of the nature of the dynamic reassortment of A(H7N9) virus with A(H9N2) virus in the environment and in poultry (*2*,*3*), close surveillance for possible new reassortment in human patients with A(H7N9) infection is needed. We report co-infection in 2 patients in Hangzhou, the capital Zhejiang Province, China, in January 2014. The co-infections involved influenza A(H7N9) virus and a seasonal A(H1N1)pdm09 virus (1 patient) or a seasonal influenza B virus (1 patient).

Of 60 patients with laboratory-confirmed influenza A(H7N9) infections in Hangzhou in April 2013 and in January–February 2014, testing of pharyngeal swab samples indicated that 2 patients were also positive for seasonal influenza virus. The pharyngeal samples were tested by real-time reverse transcription PCR according to protocols provided by the Chinese National Influenza Center. Informed consent for this study was provided by each patient’s spouse. 

On January 6, 2014, patient 1 (male, 58 years of age), a resident of Xiaoshan District, had a high fever (39.6°C) and a cough; at a hospital, he received a diagnosis of severe acute interstitial pneumonia. The patient had a history of chronic myelogenous leukemia; his history of exposure to live poultry was not clear. On January 13, infection with influenza A(H7N9) virus was laboratory confirmed; viral RNA from a pharyngeal swab sample collected before oseltamivir treatment was positive for the following: influenza A virus (cycle threshold [Ct] = 26), H7 (Ct = 27), N9 (Ct = 26), influenza A(H1N1)pdm09 virus H1 (Ct = 30), and N1 (Ct = 30). The 2 viruses were named A/Hangzhou/10–1/2014(H7N9) and A/Hangzhou/10–2/2014(H1N1)pdm09. The patient received oseltamivir while in the hospital but died on January 18.

On January 5, patient 2 (male, 54 years of age), also from Xiaoshan District, had fever and a cough; at a hospital, he received a diagnosis of severe acute pneumonia. He had a history of aplastic anemia and had been exposed to live poultry 1 week before symptom onset. On January 18, infection with influenza A(H7N9) virus was laboratory confirmed. Viral RNA from a pharyngeal swab sample collected before oseltamivir treatment was positive for the following: influenza A virus (Ct = 22), H7 (Ct = 23), N9 (Ct = 22), and influenza B virus (Ct = 22). The viruses were named A/Hangzhou/17–1/2014(H7N9) and B/Hangzhou/17–2/2014. This patient received oseltamivir but died on January 22.

The hemagglutinin (HA) and neuraminidase (NA) sequences of viruses from these 2 patients were determined by Sanger sequencing. The specific primers used are listed in Technical Appendix Table 1. The accession numbers of these sequences and the reference sequences for phylogenetic analyses are listed in Technical Appendix Table 2. Phylogenetic analyses (*4*) revealed that these 2 influenza A(H7N9) viruses were clustered into the clade of A/Shanghai/2/2013(H7N9)-like viruses (Figure). Some amino acid features within the HA and NA of these 2 viruses were the same as those in the A/Shanghai/2/2013(H7N9) strain: L226 and G228 in HA, believed to control host receptor specificity; the cleavage site in HA, relevant for virulence; a deletion in NA stalk (position 69–73), associated with the adaption to gallinaceous hosts; and R294 in NA, related to virus sensitivity to oseltamivir (*5*). The HA and NA sequences of A/Hangzhou/10–2/2014(H1N1)pdm09 and B/Hangzhou/17–2/2014 were very close to those of A(H1N1)pdm09 virus and B/Yamagata-lineage viruses that had recently circulated in China (*6*,*7*).

**Figure:**
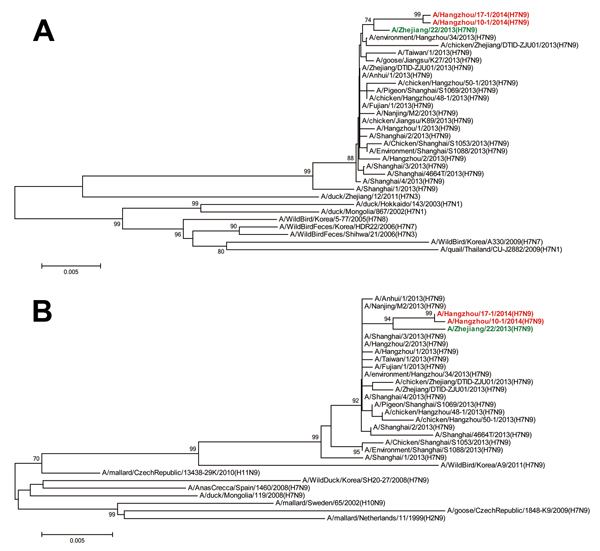
Phylogenetic analyses of hemagglutinin (A) and neuraminidase (B) of influenza A(H7N9) viruses. The trees were constructed by using the neighbor-joining method with bootstrap analysis (n = 1,000) in the MEGA5.0 program (*4*). Red indicates the 2 viruses isolated from co-infected patients in Hangzhou, China, and green indicates the first strain isolated during the second wave of the influenza A(H7N9) outbreak in China, which started in October 2013. Scale bars indicate nucleotide substitutions per site.

Co-infection with A(H7N9) virus and seasonal influenza viruses is probably associated with the overlap of A(H7N9) virus and seasonal virus circulation in both time and space and with increased prevalence of influenza virus infections within the population. From November 2012 through March 2014, outbreaks of A(H7N9) infection (in April 2013 and in January–February 2014) were concurrent with increases in seasonal influenza virus infections in Hangzhou (Technical Appendix Figure). Prompt control of A(H7N9) infection outbreaks and vaccination against seasonal influenza viruses could reduce the potential for co-infections with A(H7N9) virus and seasonal viruses.

Taken together with the previous finding of human co-infection with A(H7N9) virus and A(H3N2) virus (*1*), our results show that human co-infection with A(H7N9) virus and each of the 3 seasonal influenza viruses currently circulating worldwide can occur. Avian influenza viruses, including A(H7N9), preferentially replicate in the lower respiratory tract of humans (*8*,*9*). In contrast, seasonal influenza viruses preferentially infect the upper respiratory tract of humans (*10*). Coexistence of A(H7N9) virus with either A(H1N1)pdm09 virus or influenza B virus in the pharyngeal swab samples from 2 patients suggests that the upper respiratory tract could provide a location for the A(H7N9) virus to reassort with other influenza viruses. The possibility that seasonal influenza viruses might provide some gene segments that increase the human-to-human transmissibility of possible new reassortants is cause for concern. For detection of such new influenza virus reassortants, extensive surveillance to identify influenza virus co-infections is necessary.

Technical Appendix PCR primers used to amplify hemagglutinin and neuraminidase; GISAID or GenBank accession numbers of influenza viruses used in construction of the phylogenetic trees; and numbers of seasonal influenza virus infections and influenza A(H7N9) virus infections in Hangzhou, China, among patients with influenza-like illness at 2 sentinel hospitals.
