# Supplementary material for: Human Co-Infection with Avian and Seasonal Influenza Viruses, China
Source: Emerg Infect Dis. 2014 Nov;20(11):1953–5. doi: 10.3201/eid2011.140897 (PMC4214318; doi:10.3201/eid2011.140897)
Supplement: Technical Appendix — PCR primers used to amplify hemagglutinin and neuraminidase; GISAID or GenBank accession numbers of influenza viruses used in construction of the phylogenetic trees; and numbers of seasonal influenza virus infections and influenza A(H7N9) virus infections in Hangzhou, China, among patients with influenza-like illness at 2 sentinel hospitals. [file 14-0897-Techapp-s1.pdf]

Article DOI: http://dx.doi.org/10.3201/eid2011.140897

Human Co-Infection with Avian and Seasonal Influenza Viruses, China

Technical Appendix

Technical Appendix Table 1. Summary of PCR primers used to amplify the HA and NA genes

Gene	Primer	Primer sequence (5′→3′)	Source
A(RT)	Uni12	AGCAAAAGCAGG	(*1*)
H7 & H1	Bm-HA-1	TATTCGTCTCAGGGAGCAAAAGCAGGGG	(*1*)
	Bm-NS-890R	ATATCGTCTCGTATTAGTAGAAACAAGGGTGTTTT	(*1*)
H7	H7-H1F17	TGGTATTCGCTCTGATTGC	Designed
	H7-H1R523	TAGTCATCTGCGGGAATGC	Designed
	H7-H2F420	TAATGGAGCAACCAGTGCA	Designed
	H7-H2R944	GGACATTTTCCAACTGCCCTG	Designed
	H7-H3F834	TGGAGTACAGGTTGATGCCA	Designed
	H7-H3R1434	TTCAAAGCAACCAGTGCCA	Designed
	H7-H4F1303	TGCTGAACTCTTGGTAGCA	Designed
	H7-H4R1674	AATAGTGCACCGCATGTTTCC	Designed
N9	Bm-N9-1	TATTCGTCTCAGGGAGCAAAAGCAGGGTC	(*1*)
	Bm-N9-1473R	ATATCGTCTCGTATTAGTAGAAACAAGGGTCTT	(*1*)
	N9-N1F2	GCGAAAGCAGGGTCAAGAT	Designed
	N9-N1R501	GCTTATCAGGGCGCGATACTG	Designed
	N9-N2F416	ATGCTCTCAGCCAAGGAACAA	Designed
	N9-N2R924	TCTATTTGAGCCCTGCCAA	Designed
	N9-N3F857	GCTCATGTTACGGGGAACGA	Designed
	N9-N3R1411	TAGCCCCATCAGGCCAGTT	Designed
H1	H1R2-497	ATGAGGACATGCTGCCGTT	Designed
	H1F2-709	AGTTCAAGCCGGAAATAGC	Designed
	H1R3-1366	AGAACCAACAGTTCGGCAT	Designed
	H1F3-1262	GGGTAAAGAGTTCAACCACCTGG	Designed
	H1R5-1777	CGGAGTAGAAACAAGGGTGTT	Designed
N1	Ba-NA-1	TATTGGTCTCAGGGAGCAAAAGCAGGAGT	(*1*)
	Ba-NA-1413R	ATATGGTCTCGTATTAGTAGAAACAAGGAGTTTTTT	(*1*)
	N1R2-489	TTCGATATGGGCTCCTGTC	Designed
	N1F2-421	TGACTCAAGGGGCCTTGCTA	Designed
	N1R3-1246	TGCTGAACAAAACTCCCGCT	Designed
	N1F3-917	GAATCGACCGTGGGTGTCTT	Designed

Gene	Primer	Primer sequence (5′→3′)	Source
	N1R6-1457	TGCGTCGGAGTAGAAACAAGGAG	Designed
B(RT)	Uni11	AGCAGAAGCRS	(*2*)
B-HA	BHF1-1	TTTCTAATATCCACAAAATGAAGGCAA	Designed
	BHR1-234	GACAGTCCGGGCATAGTTTC	Designed
	B-HA-F	ATCCACAAAATGAAGGCAATA	(*3*)
	B-HA-PR	TGCAGGAGGTCTATATTGG	(*3*)
	BHF3-778	TACCACAAAGCGGCAGAATT	Designed
	BHR3-1451	TGCCAATAGATGCTCGTC	Designed
	BHF4-1285	ACCTTCAAAGACTAAGTGGTGC	Designed
	BHR6-1847	GCGCTTCAATAACGTTTCTTTG	Designed
B-NA	BNA-F1	CTGAGGCAAATAGGCCAAAAATG	(*2*)
	BNA-R1	GGGTTTAGAACAGACTCAACC	(*2*)
	BNA-280F	AAAGCACTCCTAATTAGCCC	(*2*)
	BNA-467R	GATGCCTCAGCTTGTTTCTG	(*2*)
	BNA-537F	GTCCGCATGCCATGATGGTA	(*2*)
	BNA-871R	GCTGTGTAACTGTTATCTCTACA	(*2*)
	BNF-861	AAGAATGCACATGCGGATTTGCTA	Designed
	BNR-1270	AAAGGAGTACCAACCAGGTTCTT	Designed
	BNA-1090F	TGGTACTCTCGAACGATGTC	(*2*)

Technical Appendix Table 2. GISAID or GenBank accession numbers of influenza viruses in the construction of the HA and NA phylogenetic trees

	Segments			Segments	
Viruses	HA	NA	Viruses	HA	HA
A/Hangzhou/10-1/2014(H7N9)	EPI509669	EPI509670	A/Chicken/Shanghai/S1053/2013(H7N9)	EPI440685	EPI440684
A/Hangzhou/10-2/2014(H1N1)	EPI509671	EPI511870	A/Environment/Shanghai/S1088/2013(H7N9)	EPI440693	EPI440692
A/Hangzhou/17-1/2014(H7N9)	EPI509672	EPI509673	A/Shanghai/4664T/2013(H7N9)	EPI446962	EPI446965
B/Hangzhou/17-2/2014	EPI509674	EPI509675	A/goose/Jiangsu/K27/2013(H7N9)	EPI442706	
A/Zhejiang/22/2013(H7N9)	EPI477410	EPI477412	A/chicken/Jiangsu/K89/2013(H7N9)	EPI442707	
A/Nanjing/M2/2013(H7N9)	EPI450526	EPI450527	A/duck/Zhejiang/12/2011(H7N3)	JQ906576.1	
A/Zhejiang/DTID-ZJU01/2013(H7N9)	EPI441794	EPI441797	A/duck/Hokkaido/143/2003(H7N1)	AB269694.2	
A/chicken/Zhejiang/DTID-ZJU01/2013(H7N9)	EPI442721	EPI442723	A/duck/Mongolia/867/2002(H7N1)	AB473543.1	
A/Shanghai/1/2013(H7N9)	EPI439486	EPI439487	A/WildBird/Korea/5-77/2005(H7N8)	JX444827.1	
A/Shanghai/2/2013(H7N9)	EPI439502	EPI439500	A/WildBirdFeces/Korea/HDR22/2006(H7N7)	FJ750875.1	
A/Anhui/1/2013(H7N9)	EPI439507	EPI439509	A/WildBirdFeces/Shihwa/21/2006(H7N3)	FJ767723.1	
A/Taiwan/1/2013(H7N9)	EPI445912	EPI445914	A/WildBird/Korea/A330/2009(H7N7)	JN244227.1	
A/Hangzhou/1/2013(H7N9)	EPI440095	EPI440096	A/quail/Thailand/CU-J2882/2009(H7N1)	JX523347.1	
A/Hangzhou/2/2013(H7N9)	EPI442710	EPI442711	A/WildBird/Korea/A9/2011(H7N9)		JN244234.1
A/environment/Hangzhou/34/2	EPI442716	EPI442717	A/mallard/CzechRepublic/13438-29K/2010(H1		JF789602.1

	Segments			Segments	
Viruses	HA	NA	Viruses	HA	HA
013(H7N9)			1N9)		
A/chicken/Hangzhou/48-1/2013(H7N9)	EPI443659	EPI443661	A/goose/CzechRepublic/1848-K9/2009(H7N9)		GU060482.1
A/chicken/Hangzhou/50-1/2013(H7N9)	EPI454491	EPI454492	A/AnasCrecca/Spain/1460/2008(H7N9)		HQ244409.1
A/Fujian/1/2013(H7N9)	EPI446746	EPI446747	A/duck/Mongolia/119/2008(H7N9)		AB481213.1
A/Shanghai/3/2013(H7N9)	EPI443022	EPI443023	A/WildDuck/Korea/SH20-27/2008(H7N9)		JX679164.1
A/Shanghai/4/2013(H7N9)	EPI443025	EPI443026	A/mallard/Sweden/65/2002(H10N9)		CY060360.1
A/Pigeon/Shanghai/S1069/2013(H7N9)	EPI440701	EPI440700	A/mallard/Netherlands/11/1999(H2N9)		CY122246.1

References

1. Hoffmann E, Stech J, Guan Y, Webster RG, Perez DR. Universal primer set for the full-length amplification of all influenza A viruses. Arch Virol. 2001;146:2275–89. PubMed http://dx.doi.org/10.1007/s007050170002

2. Dapat C, Saito R, Kyaw Y, Naito M, Hasegawa G, Suzuki Y, et al. Epidemiology of human influenza A and B viruses in Myanmar from 2005 to 2007. Intervirology. 2009;52:310–20. PubMed http://dx.doi.org/10.1159/000237738

3. Roy T, Agrawal AS, Mukherjee A, Mishra AC, Chadha MS, Kaur H, et al. Surveillance and molecular characterization of human influenza B viruses during 2006–2010 revealed co-circulation of Yamagata-like and Victoria-like strains in eastern India. Infect Genet Evol. 2011;11:1595–601. PubMed http://dx.doi.org/10.1016/j.meegid.2011.05.022

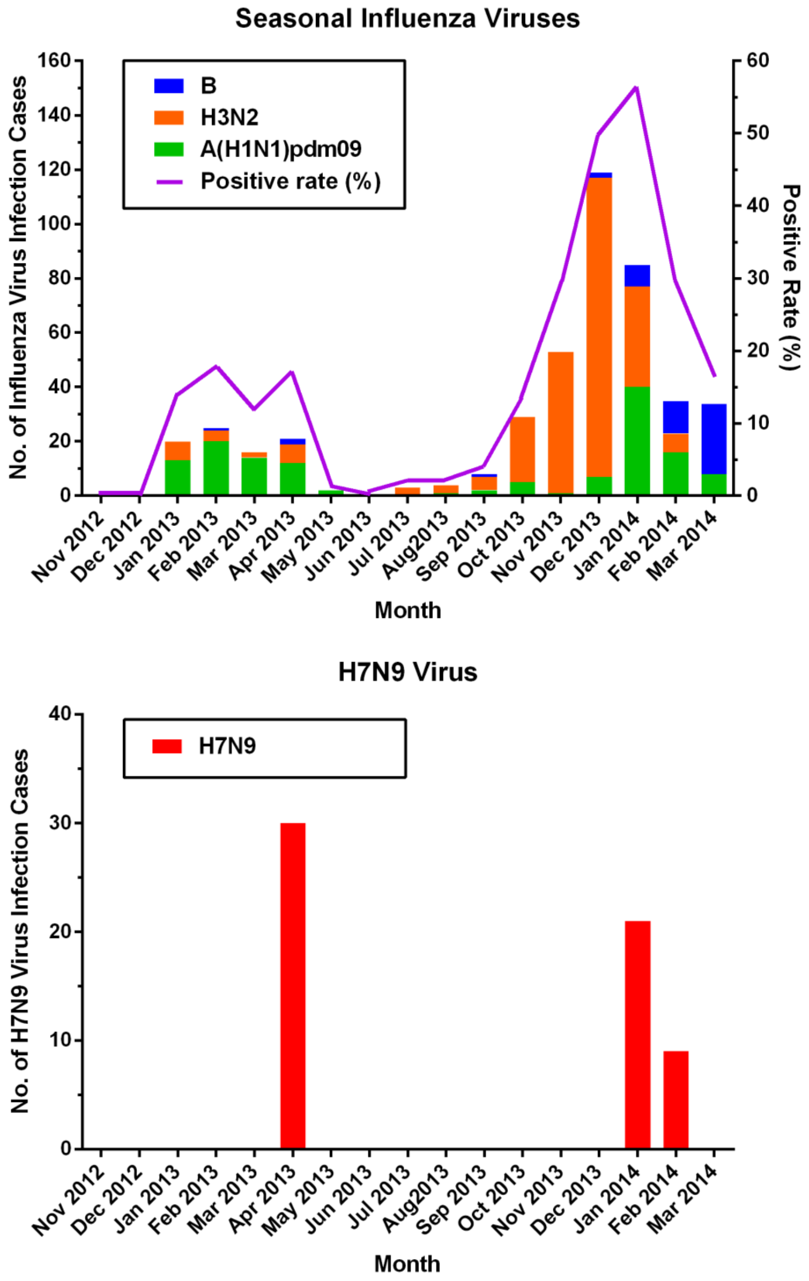

Technical Appendix Figure. Number of seasonal influenza virus infections among patients with influenza-like illness at 2 sentinel hospitals, and positivity rate, by month (upper panel) and number of influenza A(H7N9) virus infections in Hangzhou, China (lower panel). Positivity rate = no. patients with influenza-like illness who were positive for any 1 of the 3 seasonal influenza viruses/no. patients with influenza-like illness tested × 100.
